# Neurodevelopmental Effects of Propranolol Treatment During Infancy in Infantile Hemangioma Patients

**DOI:** 10.3390/children11121557

**Published:** 2024-12-22

**Authors:** Cenk Baykan, Melike Mete Çiftseven, Gonca Keskindemirci, Öykü Özbörü Aşkan, Alev Bakır Kayı, Serap Karaman, Ayşegül Ünüvar, Deniz Tuğcu, Emine Gulbin Gokcay, Merve Arslan, Zeynep Karakaş, Hikmet Gülşah Tanyıldız

**Affiliations:** 1 Department of Child Health and Diseases, Istanbul Medical Faculty, İstanbul University, 34104 Istanbul, Turkey; cenk.baykan@istanbul.edu.tr; 2 Division of Social Pediatrics, Department of Child Health and Diseases, Istanbul Medical Faculty, İstanbul University, 34098 Istanbul, Turkey; melike.mete@istanbul.edu.tr (M.M.Ç.); gonca.keskindemirci@istanbul.edu.tr (G.K.); oyku.ozboruaskan@istanbul.edu.tr (Ö.Ö.A.); alevbakirkayi@istanbul.edu.tr (A.B.K.); gulbingokcay@istanbul.edu.tr (E.G.G.); 3 Department of Social Pediatrics, Institute of Child Health, İstanbul University, 34098 Istanbul, Turkey; 4 Department of Pediatric Hematology and Oncology, Istanbul Medical Faculty, İstanbul University, 34098 Istanbul, Turkey; serap.karaman@istanbul.edu.tr (S.K.); aunuvar@istanbul.edu.tr (A.Ü.); deniz.tugcu@istanbul.edu.tr (D.T.); zkarakas@istanbul.edu.tr (Z.K.); 5 Institute of Child Health, Istanbul University, 34098 Istanbul, Turkey; merve.arslan1@medipol.edu.tr; 6 Institute of Graduate Studies in Health Sciences, Immunology, İstanbul University, 34098 Istanbul, Turkey

**Keywords:** hemangioma, propranolol, neurodevelopment, Bayley-III

## Abstract

Introduction and Aim: Propranolol is an effective treatment option for infantile hemangiomas, but there is still insufficient information about neurodevelopmental side effects of propranolol. In our study, the neurodevelopmental levels of infantile hemangioma patients receiving propranolol treatment were examined using the Bayley-III test. Method: In our single-center, cross-sectional study, patients were recruited between 1 January 2020 and 31 December 2023. In total, 40 children (n1) diagnosed with hemangioma who received propranolol treatment and 31 children (n2) who were only under observation were included. A control group of 31 healthy children (n3) matched for age and gender was also included. The demographic, clinical, perinatal, and postnatal characteristics of the total 102 children were recorded from their medical records. Neurodevelopmental levels were assessed with the Bayley-III test. The significance level was set at (*p* < 0.05). Results: The Bayley-III test composite and percentile scores were used to evaluate the neurodevelopmental levels. Significant differences in motor functions were found between the treated and untreated groups compared to the healthy control group (*p* = 0.006 and *p* = 0.006). However, no significant differences were found in cognitive, language, and social-emotional skills. Cognitive, language, and motor functions were associated with maternal education level, and additionally, cognitive functions were also associated with paternal education level. Conclusions: Propranolol has a relatively safer side effect profile, and therefore, it has been described as a safe agent. In our study, no significant effect of propranolol on neurodevelopment was observed. The difference in motor skills shown was mainly between the healthy control group (n3) and the treated and untreated group (n1 and n2), which led to the conclusion that the relevant difference could be due to factors other than propranolol itself.

## 1. Background/Rationale

Infantile hemangiomas (IHs) are the most common benign tumors of vascular origin [1,2]. Previous studies suggest that their prevalence ranges from 2% to 10%, and with advancements in medical care, the incidence of IHs is on the rise [3].

Until 2008, corticosteroids and chemotherapeutic agents were used as first-line therapy for the treatment of IHs. In 2008, Leaute-Lebreze et al. demonstrated that propranolol could also induce the involution of IHs; however, the therapeutic mechanism is still not fully understood [4]. Nonetheless, by 2012, propranolol had become the gold standard therapy for IH and is now used as a first-line therapeutic agent worldwide.

Propranolol is a non-selective, lipophilic beta-blocker used in the treatment of IHs. Because of its lipophilic nature, it can easily cross the blood–brain barrier and may cause adverse effects in the CNS. Previous animal studies have demonstrated that propranolol might cause motor dysfunctions in zebrafish larvae [5]. In adult mice, propranolol inhibits long-term potentiation and recollection of traumatic experiences via central B2 blockade, potentially prolonging the learning process [6]. An adult study conducted by Mallet et al. showed that propranolol inhibits the recollection of traumatic experiences and might reduce the severity of anxiety and depression in patients with post-traumatic stress disorder [7].

A limited number of studies have attempted to elucidate the effects of propranolol on the developing and organizing CNS. Furthermore, the use of propranolol for IH treatment has been described as the gold standard only in the last decade [8]. The primary hypothesis and objective of our study is to evaluate the potential neurodevelopmental effects of propranolol treatment in IH patients using the Bayley Scales for Infant and Toddler Development III (BSID-III) test. Our secondary objectives include assessing the factors that might influence neurodevelopment.

## 2. Methods

Our cross-sectional study was conducted in Istanbul University Istanbul Faculty of Medicine Department of Pediatrics Division of Pediatric Hematology and Oncology Clinic (PCOC) and Division of Social Pediatrics Well Child Outpatient Clinic (SP-WCOC).

In the PCOC, children with hemangioma were examined in detail for the size and number, characteristics, location and distribution of the hemangiomas. Since IHs can be seen as a part of clinical syndrome (e.g., PHACE syndrome, LUMBAR syndrome, etc.), thorough systemic physical examination is performed in order to determine the presence of accompanying findings. All children are evaluated with cranial and abdominal ultrasonography for visceral and/or intracranial hemangiomas. Complete blood count, coagulation parameters, and thyroid functions of all children are evaluated. Also, all children undergo routine electrocardiography and echocardiography for evaluation of the cardiac functions. The initiation of therapy with beta blockers, either systemically or topically, or observation-only management was determined based on treatment indications for IH, which are guided by the combined contributions of the Hemangioma Severity Score and the parents’ wishes.

In the SP-WCOC, healthy children visits are performed at fixed intervals. During each visit, children between the ages of 0 and 10 are examined and immunization profiles are assessed and screened neurodevelopmentally. Through these multidisciplinary and rigorous screening methods, children are evaluated holistically on each aspect of their wellbeing.

**Figure 1:**
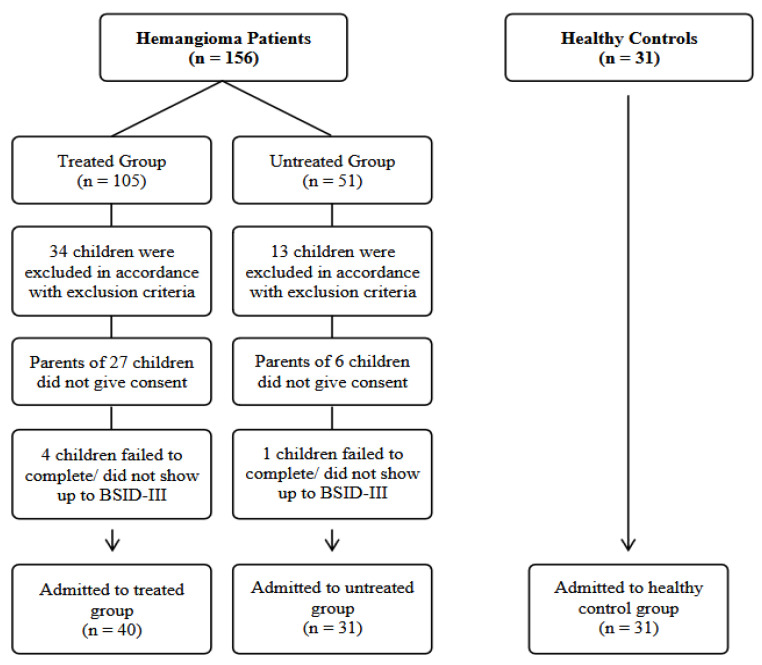
Patient recruitment flow chart. BSID-III: Bayley Scales of Infant and Toddler Development-III.

A total of 102 children were recruited between 1 January 2020 and 31 December 2023. Children were assigned to the treated hemangioma group (n1 = 40), untreated hemangioma group (n2 = 31), or the healthy control group (n3 = 31). A total of 71 hemangioma patients were recruited from the Pediatric Hematology and Oncology Department, and 31 healthy controls were recruited from the Child Health Department. The inclusion and exclusion criteria are presented in Table 1. We implemented a matching process, considering the age and gender variables of the patients to minimize selection bias. The final study size was adjusted according to the number of eligible children in both the treated and untreated groups. A flow chart of patient selection is presented in Figure 1.

A written consent form was obtained from the parents of all eligible children. Details such as age at the time of the BSID-III test, sex, mode of delivery, gestational age, admission to the Neonatal Intensive Care Unit (NICU) and the reason for admission, breast-feeding duration, and maternal and paternal education levels were noted with a pre-BSID-III test questionnaire. Additionally, information about the duration of treatment and adherence to the treatment were documented. The standard therapeutic dose of propranolol for infantile hemangioma used in our study was 2 mg/kg/day. Propranolol was initially administered at 0.5 to 1 mg/kg/day in two doses, then increased to 2 mg/kg/day in 2-week intervals.

The neurodevelopmental levels of the children who participated in the study were evaluated using the BSID-III test by a certified expert. This expert was blind to the medical records of the patients to minimize evaluation bias. The evaluation comprised four developmental sub-categories: cognitive, language, motor, and social-emotional functions, in accordance with the BSID-III test manual [9]. The BSID-III test was administered to all children in a quiet, distraction-free room under the supervision of an expert certified pedagogist, a pediatrician, and one caregiver.

After evaluation, children who were found to be at risk within any neurodevelopmental sub-categories were referred to the Pediatric Neurology and/or Children and Adolescent Psychiatry departments for further investigation.

All analyses were performed using SPSS 21.0 for Windows (IBM Corp., Armonk, NY, USA). The independent samples *t*-test was utilized to analyze the continuous variables, while chi-square (χ^2^) tests were used to compare qualitative variables. ANCOVA tests were conducted to identify co-founding factors influencing BSID-III score. *p* Values less than 0.05 were considered statistically significant.

## 3. Results

The results of the descriptive statistical analyses are presented in Table 2. Female-to-male ratios in the groups were 3:1, 1.4:1, and 1.6:1, respectively. However, the gender distribution within the cohort was homogeneous. The distribution of age at the time of BSID-III, maternal education levels, paternal education levels, and duration of breast-feeding were also homogeneous, and their respective *p*-values are presented in Table 2.

To test our hypothesis, we compared the neurodevelopmental levels of treated and untreated children with those of healthy children. These findings are summarized in Table 3. Preliminary analyses indicated a significant difference across the motor functions of the cohort, with *p*-values for motor composite and percentile scores being 0.013 and 0.005, respectively. Post hoc analyses further showed a significant difference between the treated and healthy control groups, as well as between the untreated and healthy control groups. However, post hoc analyses revealed no significant difference between the treated and untreated groups.

To further investigate the effect of propranolol on neurodevelopment, we subdivided the treated group according to the mean treatment duration in our study, which was 16 months. We divided the treated IH patients’ group into two sub-groups (n1a and n1b) based on treatment duration. We compared the BSID-III scores for these two sub-groups with those of the untreated group (n2) to analyze whether an accumulative effect of propranolol occurred. However, we found no statistical significance in any of the four neurodevelopmental categories. The results of this analysis are presented in Table 4.

The effects of other variables (e.g., maternal and paternal education levels, duration of breast-feeding, and NICU admission) on BSID-III scores were also evaluated. Preliminary analyses revealed that maternal education levels were greatly associated with infant neurodevelopment in cognitive, linguistic, and motor function categories. Post hoc analyses showed significant differences when the infant’s mother had completed at least 12 years of education compared to mothers who completed eight years or less. Paternal education levels were also associated with infant neurodevelopment, specifically in cognitive functions. Likewise, post hoc analyses showed significant differences when the infant’s father had completed at least 12 years of education compared to fathers who completed eight years or less. However, the impact of paternal education on infant neurodevelopment was found to be less substantial than that of maternal education. The results of these analyses are presented in Table 5 and Table 6.

The effects of NICU admission on BSID-III scores were analyzed. The results of BSID-III scores were compared between infants with a positive history of NICU admission (*n* = 24) and a negative history of NICU admission (*n* = 78). Analyses revealed that infants with a positive history of NICU admission had lower scores in motor functions compared to infants with a negative history of NICU admission (*p*-values for motor composite and percentile scores were *p* = 0.006 and *p* = 0.006). No statistical significance was found in cognitive, linguistic, and social-emotional categories. The results of the analyses are presented in Table 7.

The effects of confounding factors such as treatment duration, age at the start of treatment, duration of breast-feeding, and NICU admission were analyzed using m-ANCOVA tests. For the treated group, the confounding factors were exclusively the duration of treatment and the age at the beginning of treatment. On the other hand, NICU admission and the length of breast-feeding affected the entire cohort. However, no significant effect was found for any of these confounding variables. The results are presented in Table 8.

## 4. Discussion

In our study, in which we aimed to explore the potential adverse effects of propranolol, a lipophilic non-selective beta blocker drug, on the developing brain via the BSID-III test, revealed no significant neurodevelopmental effects.

In 2008, Leaute-Lebreze et al. discovered that the use of propranolol for the treatment of IHs was quite beneficial [4]. Involution of IHs with propranolol therapy was reinforced in subsequent works [10], and hence, propranolol treatment for IHs had been named as the gold standard method for the last decade and half. Considering that the first children are in the second decade of their lives as of today, it is crucial to elucidate whether the use of propranolol, especially in the first 2 years of age, has any negative effect on neurodevelopment.

Previous studies have shown that propranolol may have a negative effect on neurodevelopment and active learning, recalling traumatic experiences and long-term potentiation in animal models. Furthermore, several studies were performed to evaluate the side effects of propranolol CNS in adults, implicating conflicting results. Previous works on the effect of propranolol therapy in infantile hemangioma patients, however, were not only scarce in numbers, but also yielding inconsistent findings.

The results indicated that oral propranolol therapy at a dose of 2 mg/kg/day was not associated with significant difference in cognitive, linguistic, and social-emotional sub-categories of neurodevelopment. However, there was a significant difference in the motor functions of the children within our cohort. Post hoc analyses showed that the detected difference in motor skills was predominantly between the healthy control group (n3) and both the treated and untreated groups (n1 and n2). This led to the conclusion that the observed difference could be attributed to factors other than propranolol itself, such as parental education levels and socioeconomic status.

Furthermore, the cumulative effect of propranolol was investigated by comparing BSID-III scores of untreated children (n2) with those of infants who received propranolol for less than 16 months (n1a) and for 16 months or more (n1b). However, no statistical significance was found. Wang et al. also demonstrated that the use of propranolol for an average of 9.4 months did not cause any significant difference in IH patients compared to healthy controls [11]. We believe that the ongoing, active neuroplasticity, combined with multivariate and continuous stimuli for infants from the outside world, might have led to our inability to show any difference among groups.

A comprehensive literature review was conducted on the adverse effects of propranolol on the developing brain, yielding controversial results, particularly with regard to motor functions. In a study conducted by Wang et al., it was shown that propranolol, prescribed for complicated infantile hemangiomas (IHs), did not cause any significant neurodevelopmental delay or regression compared to healthy infants [11]. Similarly, Moyakine et al. demonstrated that the use of propranolol for the treatment of IH is not associated with gross and fine motor or psychomotor developmental defects [12,13,14]. However, Philipps et al. reported that, in their study, 146 of 188 children experienced delayed walking at 16 months of age, and significant changes in gross motor development occurred in infants with IH when propranolol treatment was stopped. This led them to raise concerns about the potential adverse effects of propranolol on the central nervous system (CNS) [15].

In our study, the level of maternal education appears to be the most significant factor impacting infant neurodevelopment in cognitive, linguistic, and motor domains. Conversely, paternal education correlated only with an infant’s cognitive functions. This difference between the effects of parental education on infant neurodevelopment might be due to unbalanced childcare responsibilities among Turkish parents. Earlier works by Ronfani et al. have demonstrated that socioeconomic status and, in particular, maternal education levels are closely and positively correlated with the cognitive and linguistic functions of infants [16]. In that same study, they found infants’ motor functions to be directly affected by maternal IQ [16]. Premkumar et al. also highlighted that parents’ educational attainment, specifically those levels equal to or greater than a high school degree, had a proportional effect on an infant’s cognitive and linguistic skills, as assessed by the BSID-III test at 18 months of age [17].

The main strength of our study lies in the incorporation of three groups: propranolol treated IHs, untreated IHs, and healthy control groups. We believe that by recruiting children into these three groups, we can better illuminate the potential adverse effects of propranolol on neurodevelopment. The BSID-III demonstrates robust and reliable validity in neurodevelopmental screening, and it also shows excellent inter-rater reliability. As far as we are aware, this study is the first to include three groups and utilize the BSID-III test concurrently.

One of the most significant limiting factors of our study is its cross-sectional design. We believe that neurodevelopment, a unique and magnificent process, occurs throughout life, suggesting that assessments of neurodevelopmental status should not be performed cross-sectionally but prospectively. Future studies should consider a broader range of variables, including demographic and socioeconomic data, to provide a more comprehensive understanding of the factors influencing neurodevelopment in this population.

## 5. Conclusions

In conclusion, our study did not demonstrate any significant developmental delays in IH patients receiving propranolol compared to untreated IH patients and healthy children. Nevertheless, we advocate for further research using a prospective design and larger samples.

## Figures and Tables

**Table 1:** Inclusion and exclusion criteria for the study.

Treated Group (n1) Inclusion Criteria	Untreated Group (n2) Inclusion Criteria	Healthy Control Group (n3) Inclusion Criteria
The use of propranolol is indicated for Infantile Hemangioma	Patient’s legal guardian’s consent for admission to the study	Patient’s legal guardian’s consent for admission to the study
Patient’s legal guardian’s consent for admission to the study		
**Treated Group (n1)** **Exclusion Criteria**	**Untreated Group (n2)** **Exclusion Criteria**	**Healthy Control Group (n3)** **Exclusion Criteria**
The use of propranolol is indicated for causes other than infantile hemangioma Presence of visceral and/or intracranial hemangiomas	The use of propranolol or any other B-Blocker either systemically or locally for infantile hemangioma Presence of visceral and/or intracranial hemangiomas	Systemic disease which may have a negative effect on neurodevelopment
Systemic disease which may have a negative effect on neurodevelopment	Systemic disease which may have a negative effect on neurodevelopment	
Gestational age < 37 weeks	Gestational age < 37 weeks	Gestational age < 37 weeks
Age at the time of BSID-III < 6 months or >42 months	Age at the time of BSID-III < 6 months or >42 months	Age at the time of BSID-III < 6 months or >42 months
Failure to obtain informed consent	Failure to obtain informed consent	Failure to obtain informed consent

Description: BSID-III: Bayley Scales of Infant and Toddler Development-III.

**Table 2:** Descriptive data of the cohort.

Variables	Treated Group (n1)	Untreated Group (n2)	Healthy Control Group (n3)	*p* Values
Sex				
Female	30	18	19	0.272
Male	10	13	12	
Age at the time of BSID-III (mo)	27.5	26	23.1	0.074
Duration of breastfeeding (mo ± SD)	16.97 ± 8.33	13.68 ± 9.3	13.22 ± 7.00	0.077
Mode of delivery				
VD	10	3	5	0.235
C/S	30	28	26	
Gestational age	39	39	39	0.825
Maternal (mo) education level				
≤8 years	17	12	7	0.177
9–12 years	12	5	8	
≥12 years	11	14	16	
Paternal education level				
≤8 years	19	12	9	0.434
9–12 years	13	9	10	
≥12 years	8	10	12	
History of NICU admission (%)	27.5	32.3	9.7	
BSID-III cognitive skills				
Composite scores (±SD)	93.75 (±15.30)	92.10 (±13.08)	99.68 (±13.22)	
Percentile value, % (±SD)	36.64 (±24.52)	35.19 (±25.17)	47.90 (±24.42)	
BSID-III language skills				
Composite scores (±SD)	93.78 (±16.74)	91.84 (±13.51)	99.59 (±13.59)	
Percentile value, % (±SD)	37.91 (±27.69)	35.10 (±24.24)	45.73 (±25.62)	
BSID-III motor skills				
Composite scores (±SD)	94.70 (±12.48)	93.13 (±11.15)	104.52 (±15.52)	
Percentile value, % (±SD)	39.83 (±24.34)	36.22 (±22.58)	55.90 (±27.53)	
BSID-III social emotional skills				
Composite scores (±SD)	105.38 (±18.36)	112.42 (±17.21)	113.68 (±21.52)	
Percentile value, % (±SD)	57.75 (±31.01)	69.02 (±27.12)	70.31 (±30.15)	

Description: SD, standard deviation; mo, months; VD, vaginal delivery; C/S, Caesarean Section; NICU, Neonatal Intensive Care Unit; BSID-III, Bayley Scales of Infant and Toddler Development-III. *p* Value for statistical significance was set at 0.05.

**Table 3:** Comparison of the BSID-III scores among all groups and post hoc analyses.

	All Groups	Treated vs. Untreated Groups	Untreated vs. Healthy Control Groups	Treated vs. Healthy Control Groups
Cognitive Composite Scores (*p* Value)	0.133	1	0.217	0.260
Cognitive Percentile Scores (*p* Value)	0.115	1	0.191	0.226
Language Composite Scores (*p* Value)	0.273	1	0.441	0.517
Language Percentile Scores (*p* Value)	0.265	1	0.409	0.531
Motor Composite Scores (*p* Value)	0.013	1	0.019	0.047
Motor Percentile Scores (*p* Value)	0.005	1	0.007	0.024
Social-Emotional Composite Scores (*p* Value)	0.102	0.339	1	0.143
Social-Emotional Percentile Scores (*p* Value)	0.135	0.506	1	0.168

Description: The “All Groups” column represents the comparison of the respective variable among all children. The 2nd, 3rd, and 4th column of the table represents the post hoc analyses of the respective variable. *p* value for statistical significance was set at 0.05. BSID-III, Bayley Scales of Infant and Toddler Development-III.

**Table 4:** Comparison of BSID-III scores of treated and untreated groups, after subgrouping according to the mean duration of time (16 months).

	All Groups
Cognitive Composite Scores (*p* Value)	0.867
Cognitive Percentile Scores (*p* Value)	0.867
Language Composite Scores (*p* Value)	0.492
Language Percentile Scores (*p* Value)	0.458
Motor Composite Scores (*p* Value)	0.885
Motor Percentile Scores (*p* Value)	0.885
Social-Emotional Composite Scores (*p* Value)	0.115
Social-Emotional Percentile Scores (*p* Value)	0.132

Description: *p* value for statistical significance was set at 0.05. BSID-III, Bayley Scales of Infant and Toddler Development-III.

**Table 5:** Comparison of BSID-III scores depending on maternal education levels.

	All Mothers	Mothers of <8 Years of Education vs. 9–12 Years of Education	Mothers of 9–12 Years of Education vs. >12 Years of Education	Mothers of <8 years of Education vs. >12 Years of Education
Cognitive Composite Scores (*p* Value)	0.001	0.108	0.762	0.001
Cognitive Percentile Scores (*p* Value)	0.001	0.087	0.866	0.001
Language Composite Scores (*p* Value)	0.038	0.815	0.719	0.031
Language Percentile Scores (*p* Value)	0.039	0.741	0.811	0.033
Motor Composite Scores (*p* Value)	0.015	0.103	1	0.017
Motor Percentile Scores (*p* Value)	0.005	0.048	1	0.007
Social-Emotional Composite Scores (*p* Value)	0.291			
Social-Emotional Percentile Scores (*p* Value)	0.128			

Description: The “All Mothers” column represents the comparison of the respective variable among all children. The 2nd, 3rd, and 4th column of the table represents the post hoc analyses of the respective variable. *p* value for statistical significance was set at 0.05. BSID-III, Bayley Scales of Infant and Toddler Development-III.

**Table 6:** Comparison of BSID-III scores depending on paternal education levels.

	All Fathers	Fathers of <8 Years of Education vs. 9–12 Years of Education	Fathers of 9–12 Years of Education vs. >12 Years of Education	Fathers of <8 Years of Education vs. >12 Years of Education
Cognitive Composite Scores (*p* Value)	0.006	0.140	0.725	0.005
Cognitive Percentile Scores (*p* Value)	0.004	0.054	1	0.005
Language Composite Scores (*p* Value)	0.071			
Language Percentile Scores (*p* Value)	0.113			
Motor Composite Scores (*p* Value)	0.057			
Motor Percentile Scores (*p* Value)	0.043	0.104	1	0.097
Social-Emotional Composite Scores (*p* Value)	0.720			
Social-Emotional Percentile Scores (*p* Value)	0.038	0.746	0.536	0.032

Description: The “All Fathers” column represents the comparison of the respective variable among all children. The 2nd, 3rd, and 4th column of the table represents the post hoc analyses of the respective variable. *p* value for statistical significance was set at 0.05. BSID-III, Bayley Scales of Infant and Toddler Development-III.

**Table 7:** BSID-III scores according to history of NICU admission.

	Negative History for NICU Admission (*n*:78)	Positive History for NICU Admission (*n*:24)	*p* Value
Cognitive Composite Scores	95 (60–145)	90 (60–115)	0.128
Cognitive Percentile Scores, %	37 (0.04–99.90)	25 (0.04–84.00)	0.123
Language Composite Scores	94 (56–141)	94 (59–121)	0.367
Language Percentile Scores, %	34 (0.02–99.70)	38 (0.03–92.00)	0.406
Motor Composite Scores	98.5 (58–145)	89.5 (64–118)	0.006
Motor Percentile Scores, %	46 (0.03–99.90)	24 (0.1–88.00)	0.006
Social- Emotional Composite Scores	110 ± 19.21	110 ± 19.44	0.930
Social- Emotional Percentile Scores, %	65.36 ± 29.71	63.82 ± 31.12	0.887

**Table 8:** Analyses of effects of co-founding variables on BSID-III scores.

	Total Duration of Breast-Feeding	History of NICU Admission	Total Duration of Treatment	Age at the Beginning of Treatment
Cognitive Composite Scores (*p* Value)	0.792	0.215	0.445	0.974
Cognitive Percentile Scores (*p* Value)	0.671	0.266	0.327	0.794
Language Composite Scores (*p* Value)	0.564	0.629	0.102	0.349
Language Percentile Scores (*p* Value)	0.588	0.897	0.057	0.145
Motor Composite Scores (*p* Value)	0.737	0.245	0.747	0.134
Motor Percentile Scores (*p* Value)	0.617	0.130	0.897	0.114
Social-Emotional Composite Scores (*p* Value)	0.616	0.273	0.198	0.679
Social-Emotional Percentile Scores (*p* Value)	0.630	0.356	0.088	0.989

Description: Two co-variants (duration of breast-feeding and History of NICU admission) were relevant for all children. Total duration of treatment and age at the beginning of treatment co-variants were exclusive for treated children. *p* value for statistical significance was set at 0.05. BSID-III, Bayley Scales of Infant and Toddler Development-III, NICU, Neonatal Intensive Care Unit.

## Data Availability

All the data in relation to this study were stored in İstanbul University, İstanbul Medical Faculty Hospital, and can be presented upon reasonable request to the corresponding author of the published work.
